# Modernizing disease assessment in prostate cancer trials: the Prostate Cancer Working Group 4 framework

**DOI:** 10.1093/oncolo/oyag251

**Published:** 2026-06-29

**Authors:** Miguel Zugman, Pedro C Barata

**Affiliations:** Centro de Oncologia e Hematologia Einstein Família Dayan—Daycoval, Hospital Israelita Albert Einstein, São Paulo, SP, 05652-900, Brazil; Division of Solid Tumor Oncology, Department of Medicine, University Hospitals Cleveland Medical Center, Cleveland, OH, 44106, United States

## Abstract

**Figure:**
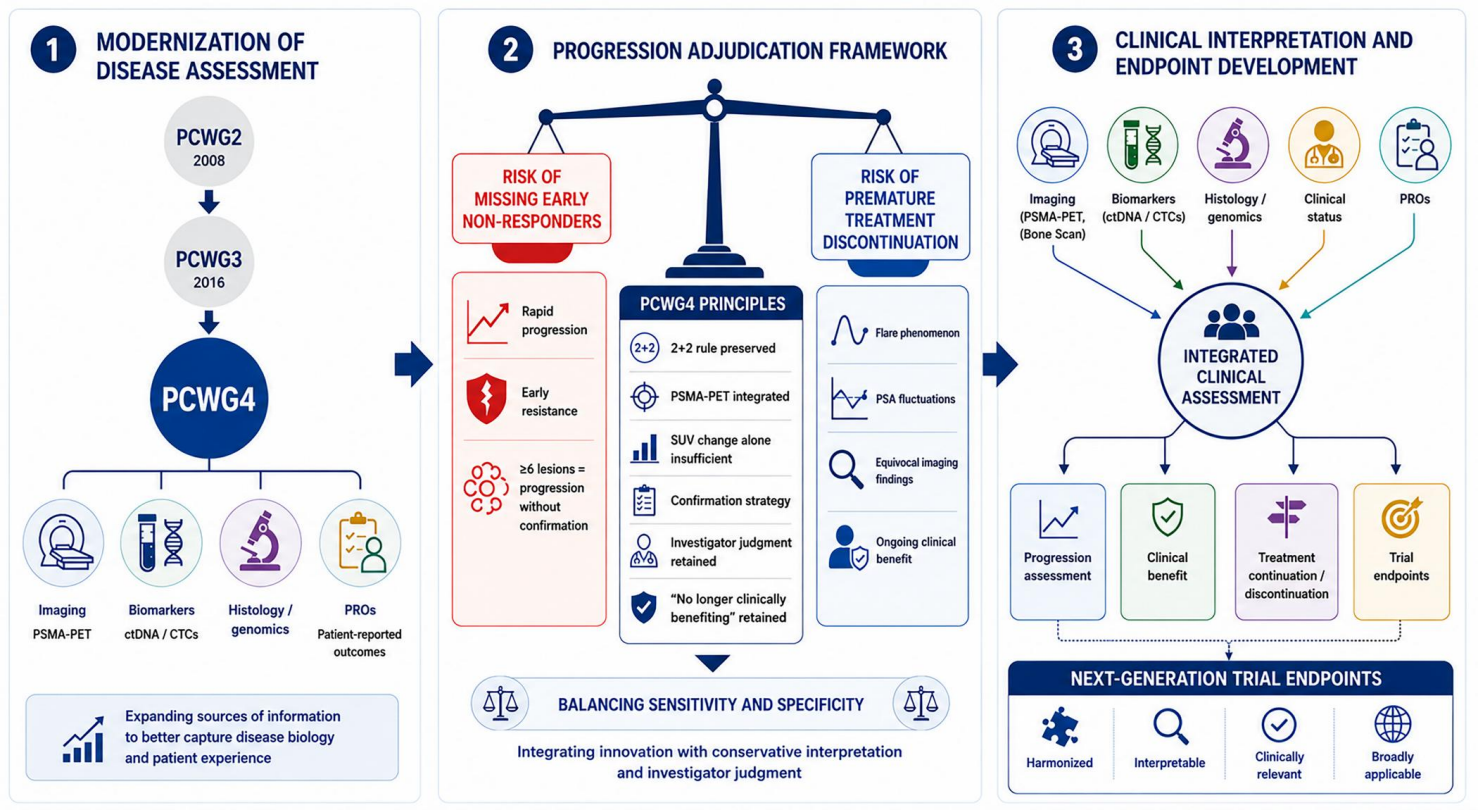

The Prostate Cancer Working Group criteria established a common vocabulary for describing and understanding prostate cancer as a disease continuum. This framework enables a shared interpretation of clinical scenarios while supporting the development of therapies that better reflect real-world clinical practice.^1^ Consequently, a central concern in applying these criteria is to balance two competing risks: (1) failing to identify patients with early, rapidly progressive disease who are not benefiting from therapy, and (2) discontinuing treatment prematurely in patients who are deriving benefit, given that early markers such as prostate-specific antigen (PSA) changes, imaging findings, or pain fluctuations may be equivocal or misleading.^2^

More recently, the advent of molecular imaging and blood-based biomarkers—including prostate-specific membrane antigen positron emission tomography (PSMA-PET) and circulating tumor DNA (ctDNA)—has reshaped how disease burden and progression are detected, challenging existing assumptions about the natural history of prostate cancer and underscoring the need to modernize disease assessment frameworks.^3^

A nomenclature update in the current framework emphasizes treatment exposure and patient experience, rather than presumed biological sensitivity. The introduction of Androgen Pathway Modulation–naïve/sensitive (APMS) and Androgen Pathway Modulation–resistant (APMR) terminology shifts the focus from disease labels to treatment exposure, recognizing that response to hormonal therapy is variable. In parallel, patient-reported outcomes are reinforced as integral components of disease assessment, with an emphasis on domain-specific, context-driven, and standardized metrics that minimize patient burden.^4^

As a major novelty, PCWG4 formally incorporates PSMA-PET, particularly for baseline assessment in localized disease, biochemical recurrence, and APMR settings, while introducing safeguards for its use in sequential disease monitoring. Consistent with PCWG3 principles, the 2 + 2 rule applies, avoiding overcalling progression and obviating the need for specialized analytic platforms, thereby ensuring broad applicability across trial sites. Changes in tracer uptake alone are not considered sufficient to define response or progression, and standardized uptake value (SUV) increase or decrease is explicitly rejected as a stand-alone criterion. There is no requirement for an 8-week new baseline scan, in contrast to bone scintigraphy, which requires precaution against flare.^5^ Therefore, comparisons are made against the pretreatment scan. As such, PSMA-PET is not used to define objective response or stable disease, as explored in other contexts.^6^

By contrast, PSMA-PET is accepted for the detection of progressive disease, particularly in trials enrolling patients whose disease is primarily identified by molecular imaging. For PSMA-avid bone, nodal, and pulmonary metastases, progression is defined by the appearance of two or more new lesions in aggregate, with confirmation on a subsequent scan at least 6 weeks later. Progression assessment should consider the combined context of uptake, size, location, and a pattern consistent with metastatic prostate cancer. In contrast, for PSMA-avid non-pulmonary visceral sites that do not meet Response Evaluation Criteria in Solid Tumors version 1.1 (RECIST 1.1) criteria—such as liver, pleural, or other visceral metastases—the appearance of any single new lesion relative to baseline constitutes progression.

Within this framework, the established 2 + 2 rule is preserved to protect against premature classification of progression, with an important refinement. The appearance of 6 or more unequivocal new lesions now constitutes progression without the need for a confirmatory scan. This applies to both bone scintigraphy and PSMA-PET (for aggregate bone, nodal, and pulmonary lesions) and is intended to improve early identification of non-responders.

The framework also expands the role of biologic characterization through systematic capture of blood-based, molecular, histologic, and genomic features, recognizing the growing heterogeneity of prostate cancer biology across disease stages and treatment contexts. Biomarker assessment is broadened beyond PSA to include ctDNA dynamics, circulating tumor cell enumeration, and somatic and germline genomic alterations, including homologous recombination repair genes, with attention to appropriate timing of collection and interpretation landmarks.^7^ Biopsies are recommended at times of treatment transition or progression, with the expectation that tissue-based information addresses predefined clinical questions with potential relevance to patient management, and/or clearly defined scientific questions that advance understanding of disease biology and preserve trial interpretability.^8^

In parallel, PCWG4 emphasizes standardized documentation of histologic subtypes and biologic phenotypes that may not align with conventional disease classifications. This includes variant morphologies such as small-cell carcinoma, neuroendocrine differentiation (*de novo* or treatment-induced), intraductal or ductal histologies, and other rare subtypes.^9^ Importantly, features reflecting potentially conflicting biology—such as aggressive-variant genomic signatures (*TP53, RB1, PTEN*), low-PSA phenotypes, FDG-avid or PSMA-discordant disease, and validated transcriptomic or digital pathology–based biomarkers—should be captured as parallel variables rather than collapsed into single categories.^10^ This approach avoids oversimplification while enabling correlative analyses across trials and platforms.

Finally, PCWG4 preserves investigator judgment as a central component of disease assessment and treatment decision-making. The framework explicitly retains the PCWG3 category of “no longer clinically benefiting”, recognizing that clinical deterioration may warrant treatment discontinuation even in the absence of formal imaging-defined progression. Conversely, the presence of radiographic or molecular progression does not mandate immediate therapy change if the investigator judges that the patient continues to derive overall clinical benefit. By maintaining a clear distinction between progression events defined for trial endpoints and decisions to alter systemic therapy, PCWG4 reinforces that no single modality should dictate clinical management in isolation.

In summary, PCWG4 advances a harmonized assessment framework across imaging modalities, biomarkers, disease sites, and assessment timepoints, while preserving the distinction between trial-defined endpoints and real-world clinical decision-making. By integrating molecular imaging, expanding biologic characterization, and reaffirming the role of investigator judgment, the framework accommodates increasing disease complexity without sacrificing interpretability or clinical relevance. In doing so, PCWG4 builds on an established foundation and provides a roadmap for the continued development of biomarkers and trial endpoints that can meaningfully inform both regulatory evaluation and patient care in the years ahead.
